# Synthesis, crystal structure and structure–property relations of strontium orthocarbonate, Sr_2_CO_4_

**DOI:** 10.1107/S2052520620016650

**Published:** 2021-01-26

**Authors:** Dominique Laniel, Jannes Binck, Björn Winkler, Sebastian Vogel, Timofey Fedotenko, Stella Chariton, Vitali Prakapenka, Victor Milman, Wolfgang Schnick, Leonid Dubrovinsky, Natalia Dubrovinskaia

**Affiliations:** a Material Physics and Technology at Extreme Conditions, Laboratory of Crystallography, University of Bayreuth, 95440 Bayreuth, Germany; b Goethe University, Institute of Geosciences, Crystallography, Frankfurt, Germany; c Department of Chemistry, University of Munich (LMU), Butendandtstrasse 513, 81377 Munich, Germany; d Center for Advanced Radiation Sources, University of Chicago, Chicago, Illinois 60637, USA; e BIOVIA Dassault Systèmes, 334 Science Park, Cambridge CB4 0WN, UK; f Bayerisches Geoinstitut, University of Bayreuth, 95440 Bayreuth, Germany; g Department of Physics, Chemistry and Biology (IFM), Linköping University, SE-581 83, Linköping, Sweden

**Keywords:** orthocarbonates, crystal structure, single-crystal X-ray diffraction, high pressure, Sr_2_CO_4_

## Abstract

Carbonates containing CO_4_ groups as building blocks have recently been discovered. A new orthocarbonate, Sr_2_CO_4_ is synthesized at 92 GPa and at a temperature of 2500 K. Its crystal structure was determined by *in situ* synchrotron single-crystal X-ray diffraction, selecting a grain from a polycrystalline sample. Strontium orthocarbonate crystallizes in the orthorhombic crystal system (space group *Pnma*) with CO_4_, SrO_9_ and SrO_11_ polyhedra as the main building blocks. It is isostructural to Ca_2_CO_4_. DFT calculations reproduce the experimental findings very well and have, therefore, been used to predict the equation of state, Raman and IR spectra, and to assist in the discussion of bonding in this compound.

## Abstract 2

A new orthocarbonate, Sr_2_CO_4_, was synthesized under extreme pressure and temperature conditions of 92 GPa and 2500 K, respectively. The crystal structure of the compound s fully characterized *in situ* by synchrotron single-crystal X-ray diffraction and DFT calculations were employed to provide insight into its equation of state, Raman and IR spectra, and bonding.

## 1. Introduction   

Carbonates have been studied extensively, from the viewpoints of both geoscience and material science [see *e.g.* Orcutt *et al.* (2019 ▸) and references therein]. In nature, the most prominent representatives at ambient conditions are two polymorphs of CaCO_3_, namely calcite and aragonite. At ambient conditions, calcite, aragonite and dolomite account for more than 90% of the natural carbonates (Reeder, 1983 ▸). Additional geologically relevant phases are dolomite [CaMg(CO_3_)_2_], magnesite (MgCO_3_) and siderite (FeCO_3_). Numerous other carbonates have been found in nature, or have been synthesized for scientific or industrial purposes. Most carbonates are either isostructual to calcite (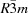), to the related structure of dolomite (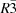) or to orthorhombic aragonite (*Pmcn*). Carbonates with large cations (cation radius > 1 Å) tend to crystallize in the orthorhombic aragonite structure type [*e.g.* cerussite (PbCO_3_), witherite (BaCO_3_) and strontianite (SrCO_3_)], while most carbonates with smaller cations tend to crystallize in the calcite or dolomite structure type {*e.g.* magnesite (MgCO_3_), dolomite [CaMg(CO_3_)_2_], siderite (FeCO_3_), rhodochrosite (MnCO_3_), otavite (CdCO_3_) and smithsonite (ZnCO_3_) [Liu & Lin (1997 ▸)]}. However there are exceptions as, for example, alkali metals form monoclinic structures [*e.g.* Li_2_CO_3_ (*C*2/*c*), K_2_CO_3_ (*C*2/*c*) and Na_2_CO_3_ (*C*2/*m*)]. In the last few years, a plethora of new carbonate phases have been discovered in high-pressure studies and complex phase diagrams have been established [*e.g.* CaCO_3_ has at least 13 polymorphs from ambient conditions to 140 GPa and 2500 K (Ono *et al.*, 2007 ▸; Ishizawa *et al.*, 2013 ▸; Lobanov *et al.*, 2017 ▸; Gavryushkin *et al.*, 2017 ▸; Bayarjargal *et al.*, 2018 ▸)]. However, until recently, it was thought that nearly planar CO_3_ groups [see Winkler *et al.* (2000 ▸) and references cited therein for a discussion on the planarity] were the defining feature of carbonates.

A remarkable discovery and a significant extension to our crystal chemical knowledge was, therefore, the synthesis and structural characterization of carbonates, in which *sp* ^3^ hybridization leads to the formation of CO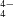 tetrahedra instead of the usual triangular *sp* ^2^-hybridized 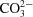 groups. The first reports of the synthesis of such novel carbonates were based on synchrotron powder X-ray diffraction and *in situ* infrared spectroscopy using either magnesite (MgCO_3_) or ferromagnesite (Mg_0.25_Fe_0.75_CO_3_) as starting compositions (Boulard *et al.*, 2011 ▸, 2015 ▸). The unequivocal experimental confirmation of carbonates with CO_4_ groups came with the utilization of single-crystal X-ray diffraction studies, where the structures of Mg_2_Fe
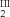
C_4_O_13_-*C*2/*c* (Merlini *et al.*, 2015 ▸), CaMg_0.6_Fe_0.4_C_2_O_6_-*Pnma* (Merlini *et al.*, 2017 ▸), Mg_2.53_Fe_0.47_C_3_O_9_-*C*2/*m* (Chariton *et al.*, 2020 ▸), Fe
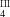
C_3_O_12_-*R*3*c* and Fe
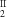
Fe
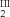
C_4_O_13_-*C*2/*c* (Cerantola *et al.*, 2017 ▸) were solved. More recently, Chariton (2020 ▸) has solved the crystal structures of MnC_2_O_5_-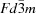 and Mn_4_C_4_O_13_-*C*2/*c*. A combination of theoretical structure predictions and Raman spectroscopy data was used to demonstrate the formation of *sp* ^3^-hybridized CaCO_3_ (Lobanov *et al.*, 2017 ▸) and MgCO_3_ (Binck *et al.*, 2020 ▸). In analogy to silicates, the CO_4_ tetrahedra may be isolated or connected to other tetrahedra by corner-sharing one or more oxygen atoms, thus forming rings, chains or pyramid-like clusters. Irrespective of the chemical composition, synthesis conditions for carbonates containing CO_4_ groups were at extreme conditions with *P* > 70 GPa and *T* > 2000 K. More recently, however, DFT calculations predicted that calcium orthocarbonate, Ca_2_CO_4_, may be formed at moderate pressures (Sagatova *et al.*, 2020 ▸). Subsequently, this prediction was verified experimentally (Laniel, 2020 ▸; Binck *et al.*, 2021 ▸) and it was found that Ca_2_CO_4_ can be formed at pressures ranging from ∼20–90 GPa.

It now seems plausible that carbonates containing CO_4_ groups can be formed with all elements for which conventional carbonates have been obtained. This would open a whole new field of crystal chemical studies, especially if it could be understood how to influence the polymerization of the tetrahedra. The present investigation supports the hypothesis of the chemical variability of carbonates with *sp* ^3^-hybridized carbon by demonstrating the formation of strontium orthocarbonate, Sr_2_CO_4_.

## 2. Experimental   

### 2.1. Synthesis and X-ray diffraction in the laser-heated diamond anvil cell   

High-pressure single-crystal X-ray diffraction experiments in a laser-heated diamond anvil cell (LH-DAC) were conducted at the P02.2 beamline at PETRA III (DESY, Hamburg, Germany). Strontium azide [Sr(N_3_)_2_] and strontium carbonate (SrCO_3_) were loaded in a BX90 diamond anvil cell (DAC) equipped with diamond anvils with 120 µm culets. The chemical precursors were prepared according to Vogel & Schnick (2018 ▸). Molecular nitrogen (N_2_) was employed as the pressure-transmitting medium. The *in situ* sample pressure was determined using the known equation of state of gold, also loaded in the sample cavity in the form of micrograins (Dewaele *et al.*, 2008 ▸). The sample was compressed to 92 GPa and laser heated to a temperature of 2500 K. Measuring the thermal radiation produced by the sample enabled the accurate determination of its temperature (Fedotenko *et al.*, 2019 ▸). Under these conditions, strontium carbonate reacted to produce strontium orthocarbonate (Sr_2_CO_4_). The produced compound was allowed to cool down to 293 K, temperature at which it was probed by X-ray diffraction. The formation of Sr_*x*_N_*y*_ compounds was also observed and will be described in an upcoming publication.

The diamond anvil cell, necessary to generate high pressures, imposes additional constraints in order to obtain high-quality single-crystal data. The high energy (λ = 0.29521 Å), small beam size (2 µm × 2 µm) and high flux of the employed P02.2 beamline of PETRAIII allow the tiny single-crystals (< 1 µm^3^) to be measured despite the intensity loss due to the scattering of the two 4 mm-thick diamond anvils. Also, the BX90 DAC used here (Kantor *et al.*, 2012 ▸) was specifically designed to maximize the angular range at which data could be collected while having sufficient mechanical stability to allow even multi-megabar pressures to be reached. It has an effective X-ray opening of −38° to +38° much larger than most DAC designs. For the vast majority of crystal structures, including that of Sr_2_CO_4_, this opening angle in combination with the high-energy X-ray wavelength allows a sufficient coverage of reciprocal space that permits an unambiguous structural solution. Still, it must be noted that the metallic body of the BX90 blocks more than 60% of all reflections, which explains the lower reflection count and 2θ range compared to ambient conditions single-crystal X-ray diffraction datasets.

In the experiments performed here, still images were recorded on a 7 × 7 grid at the center of the sample after laser heating. With this strategy, the position of the Sr_2_CO_4_ single crystal was found. A single-crystal X-ray diffraction data collection was achieved by rotating the DAC in step scans of 0.5° from −38° to +38° around the vertical axis. At each angular step, a diffraction pattern was collected with an acquisition time of 1 s.

For the data analysis, the *CrysAlis Pro* software (Rigaku, 2014 ▸) was utilized. The analysis procedure includes the peak search, the removal of the diamond anvil’s parasitic reflections and saturated pixels of the detector, finding reflections belonging to a unique single crystal, the unit-cell determination and the data integration. The crystal structures were then solved with the *SHELXT* (Sheldrick, 2008 ▸) structure solution program using intrinsic phasing and refined within the* JANA2006* software (Petříček *et al.*, 2014 ▸). The procedure for DAC single-crystal X-ray diffraction data acquisition and analysis was previously demonstrated and successfully employed (Bykova, 2015 ▸; Laniel *et al.*, 2019 ▸; Laniel, Winkler, Bykova *et al.*, 2020 ▸; Laniel, Winkler, Fedotenko *et al.*, 2020 ▸).

### 2.2. Refinement   

Crystal data, data collection details and structure refinement details are summarized in Table 1 ▸. As these measurements were performed in a DAC, the angular range over which single-crystal data is available is limited. For this reason, the data resolution was insufficient to anisotropically refine the atomic displacement parameters (ADP) of all atoms. Hence, anisotropic displacement parameters were refined only for the strontium atoms, while for the oxygen and carbon atoms the refinement was restricted to isotropic ADP. Due to the synthesis method of Sr_2_CO_4_, nitrogen may have been incorporated into the crystal structure. However, as no significant residual electronic density at chemically relevant distances remains in the crystal, the incorporation of nitrogen is implausible. Moreover, we tested for an unlikely substitution of either carbon or oxygen with nitrogen. An increase in *R*-factors was observed when performing the substitution of C
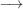
N (Δ*R*1 = 0.034) or O
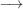
N (Δ*R*1 = 0.013 to 0.033, depending on the substituted O atom). Therefore is no indication of the presence of nitrogen in the crystal structure.

### 2.3. Density functional theory-based calculations   

Density functional theory (DFT) calculations have been performed using the CASTEP code (Clark *et al.*, 2005 ▸). The code is an implementation of Kohn–Sham DFT based on a plane wave basis set in conjunction with pseudopotentials. The plane wave basis set allows numerically converged results in a straightforward manner to be achieved, as the convergence is controlled by a single adjustable parameter, the plane wave cut-off, which was set to 1020 eV. The norm-conserving pseudopotentials were generated on the fly from the information provided in the CASTEP data base. These pseudopotentials have been tested extensively for accuracy and transferability (Lejaeghere *et al.*, 2016 ▸). All calculations employed the GGA-PBE exchange-correlation functional (Perdew *et al.*, 1996 ▸). The Brillouin zone integrals were performed using Monkhorst–Pack grids (Monkhorst & Pack, 1976 ▸) with spacings between grid points of less than 0.037 Å^−1^. Geometry optimizations were defined as being converged when the energy change between iterations was < 0.5 × 10^−6^ eV per atom, the maximal residual force was < 0.01 eV Å^−1^, and the maximal residual stress was <0.02 GPa. Phonon frequencies were obtained from density functional perturbation theory (DFPT) calculations. Raman intensities were computed using DFPT in the 2*n* + 1 theorem approach (Miwa, 2011 ▸).

## 3. Results and discussion   

### 3.1. Experimental crystal structure of Sr_2_CO_4_ at 92 GPa   

**Figure 1:**
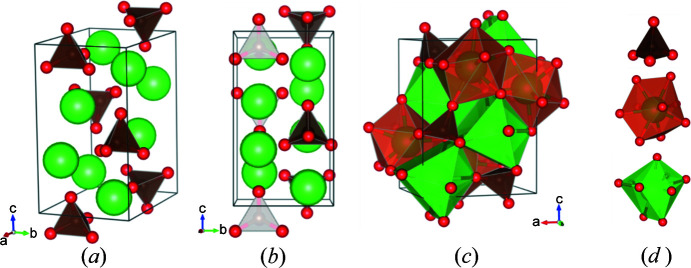
(*a*) Crystal structure of the Sr_2_CO_4_ orthocarbonate at 92 GPa. (*b*) Viewed along the *a* axis, when the atoms lying on the *a* *c* mirror plane (*b* = 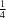 and 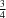) are clearly visible. (*c*) Polyhedral representation of Sr_2_CO_4_. (*d*) The three building blocks of Sr_2_CO_4_, namely: CO_4_, SrO_11_ and SrO_9_ (top to bottom).

Strontium orthocarbonate, Sr_2_CO_4_ crystallizes in the orthorhombic crystal system with space-group symmetry *Pnma*. At 92 GPa, the unit-cell parameters were determined to be *a* = 6.214 (12), *b* = 4.6353 (14) and *c* = 8.083 (2) Å [*V* = 232.8 (5) Å^3^]. The crystal structure is shown in Fig. 1 ▸ and Table 1 ▸ contains selected crystal data. Eight distinct atoms compose the structure with all, except one oxygen atom (O1), occupying the 4*c* special Wyckoff position which lies on the *a* *c* mirror plane with *b* = 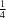 and 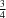. The O1 oxygen atom rests on the 8*d* general position. The atomic arrangement gives rise to three types of coordination polyhedra: CO_4_, SrO_9_ and SrO_11_. At 92 GPa, the CO_4_ tetrahedra share corners, edges and faces with the SrO_11_ polyhedra, but only share corners and edges with the SrO_9_ polyhedra. The SrO_9_ and SrO_11_ polyhedra are connected to each other via their faces. While the SrO_9_ polyhedra are of irregular shape, the SrO_11_ polyhedra form pentacapped trigonal prisms. The CO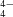 group has four C—O bonds with lengths of 1.31 (5), 1.37 (2), 1.37 (2) and 1.38 (3) Å, and bond angles that vary between 103.2 (18) and 120 (3)°. These values are consistent with those previously reported for carbonates with *sp* ^0^-hybridized carbon (Chariton *et al.*, 2020 ▸; Binck *et al.*, 2021 ▸). The SrO_9_ and SrO_11_ polyhedra have an average Sr—O distance of 2.42 (2) and 2.27 (1) Å, respectively, with a minimum and maximum contact length of 2.203 (13) and 2.495 (11) Å, respectively.

### 3.2. DFT calculations   

At 92 GPa, the pressure at which Sr_2_CO_4_ was synthesized here, the experimentally derived structural model is well reproduced by DFT calculations (Table 1 ▸). The satisfactory reproduction of the experimentally determined structural parameters by DFT model calculations allows us to confidently predict properties and to investigate structure–property relations.

#### 3.2.1. Bonding in orthocarbonates   

**Figure 2:**
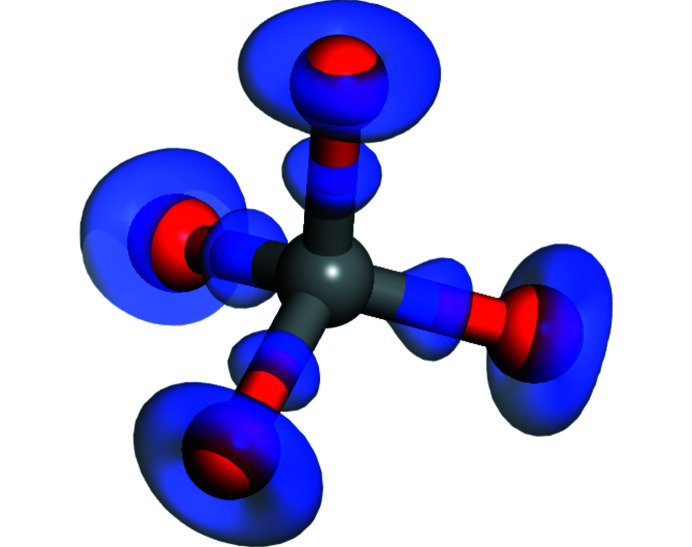
Isosurface of the electron density difference. The isosurface is plotted for a value of 0.2 e Å^−3^ and shows those regions in which the electron density, after reaching self-consistency, is larger than the electron density obtained by overlapping electron densities of non-interacting atoms. Clearly, there is charge accumulation halfway along each of the four C—O vectors, which is indicative of the formation of covalent bonds.

A Mulliken population analysis shows that the three symmetrically independent C—O bonds are very similar: at 1 bar (1 bar = 10^5^ Pa) the bond population decreases slightly from 0.7 e Å^−3^ for the shortest bond to 0.6 e Å^−3^ for the longest bond. A plot of the electron density difference confirms this. In such a plot (Fig. 2 ▸), the difference between the self-consistent electron density and the density obtained by overlapping the electron density of non-interacting atoms is shown.

Clearly, in the CO_4_ group there are four very similar covalent C—O bonds. It is instructive to compare the CO_4_ groups to those of SiO_4_ groups in an isostructural Sr_2_SiO_4_ silicate. The C—O bonds are, as expected, shorter (≃1.4 Å compared to 1.63 Å for the Si—O bond in the silicate) but the bond populations are very similar (≃0.65 e Å^−3^) in both compounds. Another notable difference is the Mulliken charge of Si^4+^ to C^4+^, where the former is 1.6 e and the latter is only 0.55 e. The Mulliken charge of Sr^2+^ is, in both compounds, ≃1.5 e, and consequently the Mulliken charge of the O^2−^ is notably less in Sr_2_CO_4_ (−0.9 e) than in isostructural Sr_2_SiO_4_, where it is −1.16 e. So, while there are some crystal chemical similarities, the formation of solid solutions in which SiO_4_ groups are substituted by CO_4_ groups is unlikely, especially as the volume of the former is about twice that of the latter (Milman *et al.*, 2001 ▸).

#### 3.2.2. Compression of orthocarbonates   

**Figure 3:**
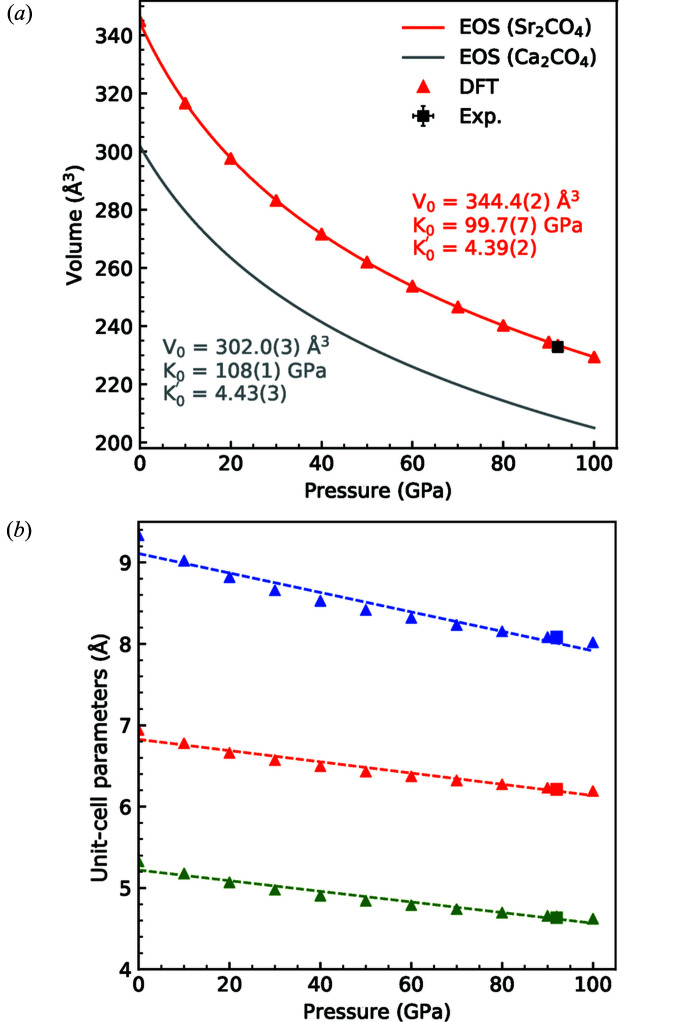
(*a*) Pressure–volume data of Sr_2_CO_4_ (this study) and Ca_2_CO_4_ (Binck *et al.*, 2021 ▸) between 1 bar and 100 GPa. The data is fitted with a third-order Birch–Murnaghan equation of state yielding *K*_0_ = 99.7 (7) GPa, *K*_0_′ = 4.39 (2) and *V*_0_ = 344.4 (2) Å^3^. (*b*) Evolution of the unit-cell parameters of Sr_2_CO_4_. The red, green and blue symbols refer to the *a*, *b* and *c* unit-cell parameters, respectively.

**Figure 4:**
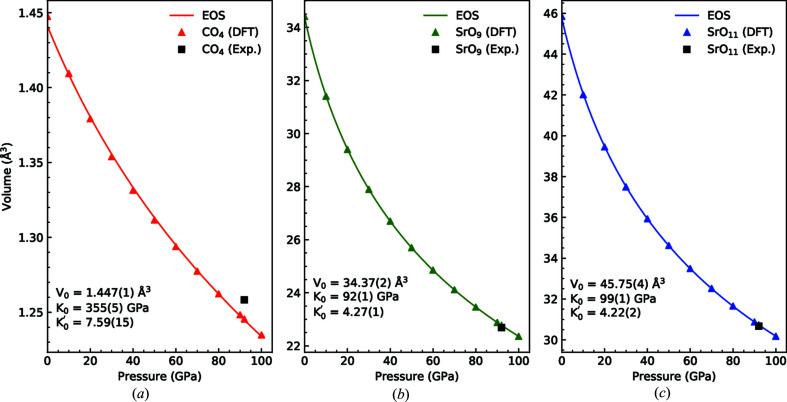
Pressure–volume evolution of the three building blocks of Sr_2_CO_4_: CO_4_ (*a*), SrO_9_ (*b*) and SrO_11_ (*c*). As expected from its short and rigid C—O single bonds the CO_4_ tetrahedron is found to be very incompressible.

Fig. 3 ▸ shows the fit of a third-order Birch–Murnaghan equation of state (Birch, 1947 ▸) to the Sr_2_CO_4_ *P*–*V* data. From this, a bulk modulus of *K*_0_ = 99.7 (7) GPa was obtained, with a pressure derivative of *K*_0_′ = 4.39 (2) and an ambient-pressure volume of *V*_0_ = 344.4 (2) Å^3^. In a similar fashion, the change of volume with pressure for the three building blocks of Sr_2_CO_4_, namely CO_4_, SrO_9_ and SrO_11_, were calculated and are shown in Fig. 4 ▸. As expected from its four rigid C—O single bonds, the CO_4_ tetrahedron is found to be very incompressible [*K*_0_ = 355 (5) GPa], while the SrO_9_ and SrO_11_ display a much lower value of *K*_0_ = 92 (1) GPa and *K*_0_ = 99 (1) GPa, respectively.

Strontium orthocarbonate, Sr_2_CO_4_ is isostructural to calcium orthocarbonate, Ca_2_CO_4_ (Sagatova *et al.*, 2020 ▸; Laniel, 2020 ▸; Binck *et al.*, 2021 ▸). The comparison of the unit-cell parameters of Ca_2_CO_4_ and Sr_2_CO_4_ shows the expected influence of the cation substitution as the unit-cell volume increases by about 14% when Ca^2+^ is substituted by Sr^2+^ at ambient pressure. At 100 GPa, the difference between the unit-cell volume is similar (12%).

The isothermal bulk modulus of Ca_2_CO_4_ [*K*_0_ = 108 (1) GPa] is 8% larger than that of Sr_2_CO_4_ [*K*_0_ = 99.7 (7) GPa] (Fig. 3 ▸). This is similar to the relation of the compressibility of aragonite [*K*_0_ = 69 (1) GPa] and strontianite [*K*_0_ = 62 (1) GPa]. The CO_4_ tetrahedra in both Sr_2_CO_4_ and Ca_2_CO_4_ are very incompressible [for Sr_2_CO_4_: *K*_0_ (CO_4_) = 355 (5) GPa, for Ca_2_CO_4_: *K*_0_ (CO_4_) = 360 (38) GPa], even compared to SiO_4_ tetrahedra [*K*_0_ (SiO_4_) = ∼300 GPa] (Binck *et al.*, 2020 ▸).

#### 3.2.3. Lattice dynamics of orthocarbonates   

It is very well established that DFPT calculations can reliably predict Raman spectra once the underlying structural model is established. For CaCO_3_ and MgCO_3_ polymorphs this has been demonstrated by Bayarjargal *et al.* (2018 ▸) and Binck *et al.* (2020 ▸), respectively. Raman spectra for SrCO_3_ polymorphs have been published by Biedermann *et al.* (2017 ▸). No Raman spectra of Sr_2_CO_4_ have been obtained yet, but high-quality data are available for isostructural Ca_2_CO_4_ (Binck *et al.*, 2021 ▸).

**Figure 5:**
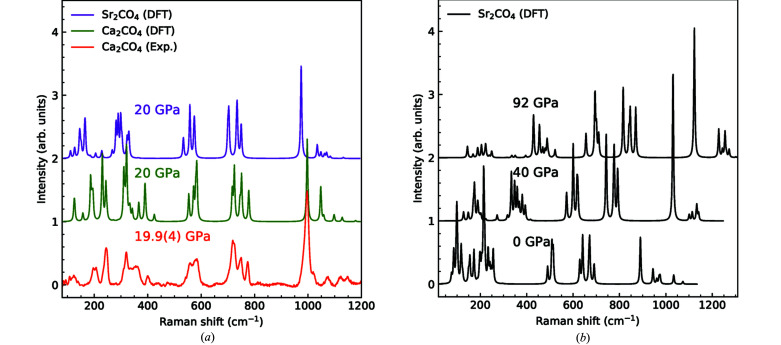
(*a*) Comparison of a DFT-calculated Raman spectrum of Sr_2_CO_4_ (purple, this study), to DFT-calculated (green) and experimental (red) Raman spectra of Ca_2_CO_4_ at ∼20 GPa as obtained by Binck *et al.* (2021 ▸). (*b*) DFT-calculated Raman spectra of Sr_2_CO_4_ at different pressures. The shift of Raman modes towards higher frequencies implies positive Grüneisen parameters for all modes. All DFT-calculated Raman spectra have their *x* axis (Raman shift) multiplied by a scaling factor of 4%.

The group theoretical analysis for Sr_2_CO_4_ is the same as for Ca_2_CO_4_. Both crystallize in the centrosymmetric space group *Pnma*, so the Raman active modes cannot be IR active and vice versa. The unit cells of these compounds contain *n* = 28 atoms each. Of the 3*n* = 84 modes, 42 are Raman active. Three of the 34 IR-active modes are acoustic phonons and cannot be measured. A group theoretical analysis gives Γ_Raman_ = 13A_g_ + 8B_1g_ + 13B_2g_ + 8B_3g_ and Γ_IR_ = 12B_1u_ + 7B_2u_ + 12B_3u_ (acoustic modes not included). In Fig. 5 ▸(*a*), we compare the predicted Raman spectrum of Sr_2_CO_4_ to experimental and DFT data for Ca_2_CO_4_ at 20 GPa.

**Figure 6:**
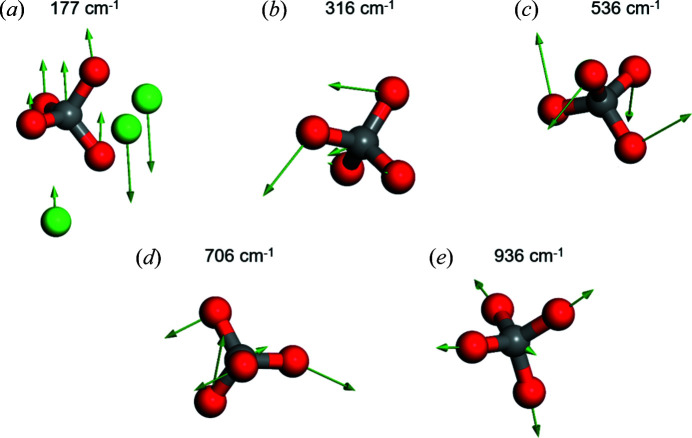
Displacement patterns in typical Raman modes of Sr_2_CO_4_ at 20 GPa. Arrows indicate the displacement of the atoms during the specific vibration. Low-frequency modes [*e.g.* (*a*)] are dominated by relative motions of the CO_4_ groups against the Sr ions. Intermediate frequencies [*e.g.* (*b*)] are mainly due to displacements/rotations of the CO_4_ groups, while the Sr ions are at rest. Raman shifts > 500 cm^−1^ [(*c*), (*d*) and (*e*)] are due to various bending and stretching vibrations in the CO_4_ groups, while the Sr ions are at rest.

As expected, the Raman spectra of Sr_2_CO_4_ and Ca_2_CO_4_ [Figs. 5 ▸(*a*) and 5 ▸(*b*)] are very similar. We use the DFT data to identify the dominant atomic displacements in the characteristic vibrations. Typical displacement patterns are shown in Fig. 6 ▸.

As the phonons with wavenumbers > 500 cm^−1^ are dominated by modes in which only the CO_4_ groups are deformed, the Raman shifts of Sr_2_CO_4_ and Ca_2_CO_4_ are very similar in that region. Only at lower frequencies are the Raman shifts in Sr_2_CO_4_ red-shifted with respect to those in Ca_2_CO_4_ due to higher mass of Sr^2+^ with respect to Ca^2+^. The predicted pressure dependence of the Raman spectra is shown in Fig. 5 ▸, which can now be used to identify Sr_2_CO_4_ and carbonates containing CO_4_ groups in high-pressure experiments.

## 4. Conclusion   

The present study has expanded our knowledge of carbonates containing CO_4_ groups by adding Sr_2_CO_4_ to this family of compounds. The crystal structure of strontium orthocarbonate, Sr_2_CO_4_, was unambiguously determined using single-crystal X-ray powder diffraction measurements. It was found to be isostructural to another orthocarbonate, Ca_2_CO_4_. The present study has shown yet another way on how to synthesize carbonates with *sp* ^3^-hybridized carbon by a more complex chemical reaction than has been employed in earlier studies. We have used the example of Sr_2_CO_4_ to discuss the bonding in this fascinating class of compounds and have identified characteristic features in the lattice dynamics, thus, facilitating the identification of *sp* ^3^-carbon in carbonates by Raman spectroscopy. The pressure stability range of Sr_2_CO_4_ and the conditions under which it can be formed have not been explored yet. Such experiments are currently underway.

## Supplementary Material

CCDC reference: 2052128

## Figures and Tables

**Table 1:** Crystal data on the Sr_2_CO_4_ compound for single-crystal X-ray diffraction measurements performed at 92 GPa

Crystal data	
Chemical formula	Sr_2_CO_4_
*M*_r_	251.2
Crystal system, space group	Orthorhombic, *Pnma*
Temperature (K)	293
*a*_exp_, *b*_exp_, *c*_exp_ (Å)	6.214 (12), 4.6353 (14), 8.083 (2)
*a*_DFT_, *b*_DFT_, *c*_DFT_ (Å)	6.2223, 4.6497, 8.687
*V*_exp_ (Å^3^)	232.8 (5)
*V*_DFT_ (Å^3^)	233.4
*Z*	4
Radiation type, wavelength (Å)	Synchrotron, 0.29521
μ (mm^−1^)	4.31
Crystal size (mm)	0.001 × 0.001 × 0.001

Data collection	
Diffractometer	Esperanto-*CrysAlis PRO*-abstract goniometer imported esperanto images on P02.2 at PETRA III
Absorption correction	Multi-scan (*CrysAlis PRO*). Empirical absorption correction using spherical harmonics, implemented in SCALE3 ABSPACK scaling algorithm.
*T*_min_, *T*_max_	0.339, 1
No. of measured, independent and observed [*I* > 3σ(*I*)] reflections	676, 230, 170
*R*_int_	0.054
(sin θ/λ)_max_ (Å^−1^)	0.887

Refinement	
*R*[*F* ^2^ > 2σ(*F* ^2^)], *wR*(*F* ^2^), *S*	0.045, 0.051, 2.48
No. of reflections	230
No. of parameters	26
Δρ_max_, Δρ_min_ (e Å^−3^)	2.23, −1.55

Computer programs: *CrysAlis PRO* 1.171.40.55a (Rigaku Oxford Diffraction, 2019 ▸).
